# Psychological Impact and Associated Factors During the Initial Stage of the Coronavirus (COVID-19) Pandemic Among the General Population in Spain

**DOI:** 10.3389/fpsyg.2020.01540

**Published:** 2020-06-23

**Authors:** Rocío Rodríguez-Rey, Helena Garrido-Hernansaiz, Silvia Collado

**Affiliations:** ^1^Department of Psychology, Faculty of Humanities and Social Sciences, Universidad Pontificia Comillas, Madrid, Spain; ^2^Department of Education and Psychology, Centro Universitario Cardenal Cisneros, Alcalá de Henares, Spain; ^3^Department of Psychology and Sociology, Faculty of Social and Human Sciences, Universidad de Zaragoza, Teruel, Spain

**Keywords:** COVID-19, pandemic, psychological impact, psychological crisis intervention, stress, anxiety, depression

## Abstract

The outbreak of COVID-19 in Spain started at the end of February. By 9th April 2020 Spain was the second country in confirmed cases and in deaths. On March 14, 2020, the Spanish Government declared the state of alarm to limit viral transmission. During such state, citizens must stay confined at home with few justified exceptions. This whole situation drastically changed the life of the population, which can cause a wide range of psychosocial impacts. This study explored the psychological impact of the COVID-19 pandemic in the general adult population (*N* = 3055) during the first stages of the outbreak in Spain, as well as their anxiety, stress and depression levels. We also examined the extent to which the following variables were associated to participants’ mental health: (1) demographics; (2) degree of concern about the pandemic; (3) environmental conditions during the home confinement, (4) changes in daily life as a consequence of the pandemic; (5) contact with the COVID-19 disease; (6) actual and perceived severity of the crisis; (7) information about the COVID-19, (8) perceived health status and (9) leisure activities conducted within the last 24 h. Our results show that Spanish consider the current COVID-19 health crisis as fairly severe, and the majority felt that the COVID-19 crisis had greatly impacted on their daily life, including changes in their daily routines and cancelation of important activities. About 36% of the participants reported moderate to severe psychological impact, 25% showed mild to severe levels of anxiety, 41% reported depressive symptoms, and 41% felt stressed. Women, young, and those who that lost their job during the health crisis showed the strongest negative psychological symptoms. What worried Spaniards the most was the likelihood of suffering an economic crisis derived from the pandemic. We found factors associated with better mental health, such as being satisfied with the information received about the health crisis, conducting leisure activities, and the perception of being in good health. These findings can be used to design psychological interventions to help coping with COVID-19 pandemic, both in Spain and other countries.

## Introduction

In December 2019, the new coronavirus disease 2019 (COVID-2019) started spreading in the Chinese city of Wuhan (Hubei province). The most typical symptoms of the disease are fever, myalgia, fatigue, and dry cough. Other referred symptoms are chills, coryza, sore throat, nausea, vomiting, and diarrhea (Chen et al., 2020; Huang et al., 2020). These symptoms are usually mild, and some infected people are asymptomatic (Rothe, 2020; Ryu et al., 2020). According to the World Health Organization (World Health Organization, 2020), about 80% of infected people easily recover from COVID-19, without the need of any specific treatment. However, about 1 out of 6 cases of infection courses with severe pneumonia (Bermejo-Martin et al., 2020), which can lead to respiratory failure, cardiac injury, acute respiratory distress syndrome and death (Holshue, 2020). COVID-19 virus spreads from person to person via virus-laden respiratory droplets produced when an infected person talks, coughs, exhales or sneezes. These droplets can be inhaled by the people nearby, and/or fall over objects and surfaces, which another person can touch, and then touch their nose, eyes or mouth and get infected (World Health Organization, 2020; Centers for Disease Control and Prevention, 2020). COVID-19 is considered a highly contagious virus (Yang and Wang, 2020). Thus, even though only a minority of infected people develop severe symptoms, COVID-19 is a global health threat. In fact, on the 30 January 2020, the WHO declared the health outbreak caused by COVID-19 a public health emergency of international concern. Considering its rapid spread, it is not surprising that the first cases of infected people in Europe were reported only a few weeks after. The first transmission was reported in Italy, on February 21st 2020, and it soon became the largest COVID-19 outbreak outside Asia (Spina et al., 2020). Shortly after, by the end of February, the outbreak started in Spain. On March 11 2020, the WHO upgraded the status of the COVID-19 outbreak from epidemic to pandemic. According to official data (European Centre for Disease Prevention and Control, 2020), by April 9th 2020, there were nearly 1.5 million cases worldwide and over 87,000 deaths. The majority of infected people live in the United States. Spain is the second country in confirmed cases (146,690) and the third in deaths (14,555). As of April 3rd, the number of daily new cases in Spain per day seem to have stabilized and even begun to decrease, although the number of active cases is still increasing. This is mainly due to the severe movement restrictions taken by the Spanish Government in order to mitigate the spread. This unusual situation of health emergency and the social restrictions taken to control the COVID-19 spread are likely to have negative consequences on Spaniards mental health (Wang et al., 2020; Xiang et al., 2020). However, there is a lack of information regarding the psychological impact of the COVID-19 pandemic in the general Spanish population. The aim of the present study is to fill this gap in the literature.

As indicated, different measures have been taken in Spain to mitigate the virus spread. One of the first measures taken (between the 11 and the 13 of March depending on the Spanish province) was canceling every on-site educational activity from kindergarten to the University. Shortly after, on March 14, 2020, the Spanish Government declared the state of alarm, which came into effect the following day when extraordinary measures were implemented to limit viral transmission at a national level. The state of alarm was first declared until the 29 of March, and then extended twice, the first time until April 11, and the second until April 26. During the state of alarm, citizens are allowed to be on public roads and streets only for the purchase of essential items (e.g., food, medicines), attend health centers, go to work (only for jobs considered essential, such as food suppliers), return to the usual residence, assist and care for dependants and other cases of force majeure.

This whole situation has drastically changed the life of people living in Spain in a matter of days. The population is experiencing a new, unpredictable and rapidly evolving situation. They have to stay confined at home, family dynamics have remarkably changed, travel is restricted, and there has been a reduction in leisure activities and social life. The work situation has also changed thoroughly; many people have temporary or permanently lost their jobs, many are working from home, sometimes with insufficient preparation for doing so, and those who work in sectors considered essential appear to experience heavy workloads, increased levels of stress and a greater exposure to the virus. The Spanish health system has been overwhelmed and there have been shortages of space in the hospitals (mostly in the Emergency rooms and in the Intensive Care Units, UCIs), of health equipment (mostly ventilators) and of personal protective equipment (PPE). This situation seems to be lived with a high level of fear and concern about the pandemic and its consequences. In fact, in Wuhan, residents compared this health crisis with “the end of the world” (Lima et al., 2020).

The majority of the research conducted about COVID-19 relates to its clinical characteristics (Xu et al., 2020), likelihood of survival (Ruan, 2020), genomic characterization of the virus (Lu et al., 2020) and drug and therapeutic options (Al-Tawfiq et al., 2020). Significantly less scientific efforts have been placed into analyzing the psychological impact of COVID-19 pandemic. Moreover, given that the outbreak started in China, the scarce literature about the psychological consequences of this global health crisis relate to Chinese population. According to Xiang et al. (2020), patients with confirmed COVID-19 or with compatible symptoms may experience fear of the consequences of the disease, and some symptoms, such as fever or shortness of breath can aggravate mental distress and anxiety. In addition, the unpredictability of the current crisis, and the misinformation derived from it makes the whole situation more stressful (Bao et al., 2020). These psychological difficulties to cope with the current situation are aggravated with the extreme measures taken by the Governments of different countries to ameliorate the virus spread, especially by keeping people in quarantine. According to the recent review conducted by Brooks et al. (2020), being forced to stay at home leads to negative psychological effects such as fear, frustration and anger. The negative impact of the confinement can have long-lasting effects. In line with this review, people in China experienced boredom, loneliness and anger while being confined, as well as an increase in psychological problems, such as anxiety, stress and depression (Duan and Zhu, 2020). In such a difficult context, many authors recognize that taking care of the population’s mental health is essential (Brooks et al., 2020; de Carvalho et al., 2020; Duan and Zhu, 2020; Zandifar and Badrfam, 2020), and that more research is needed in different parts of the world to fully understand the negative psychological consequences of the pandemic and, accordingly, formulate psychological intervention to mitigate them (Xiang et al., 2020).

Studies related to previous outbreaks such as Ebola, swine flu or MERS have revealed that such situations cause a deep and wide range of negative psychosocial impacts. Common psychological reactions are fear of contracting the virus and falling sick (Rubin et al., 2010; Al Najjar et al., 2016), of dying, of suffering if being infected, of separation from relatives and stigma, as well as feelings of helplessness (Hall et al., 2008). Such negative emotions tend to intensify with the restrictions usually taken by the authorities to ameliorate the virus spread, such as closure of schools and business (Van Bortel, 2016). For instance, during the Middle East respiratory syndrome-novel coronavirus outbreak (MERS CoV) in Jeddah (Western Saudi Arabia), 57.7% of interviewed people reported moderate levels of anxiety (Al Najjar et al., 2016). In an effort to understand mental health status of the Chinese general population during the early stage of COVID-19 outbreak, Wang et al. (2020) conducted a cross-sectional study with a sample of more than 1,000 adults. In line with Al Najjar et al.’s (2016) findings, 53.8% of the participants reported a moderate to severe psychological impact. The authors also registered depression, anxiety, and stress levels derived from health emergency. Considering depression, 13.8% reported mild depression symptoms, 12.2% were considered to suffer from moderate depression, and 4.3% from severe depression. Chinese also suffered from anxiety (7.5% mild, 20.4% moderate and 8.4% severe). In addition, 24.1% reported suffering from mild stress while 8.1% reported moderate or severe stress levels. Sun et al. (2020) explored the prevalence and risk factors of acute posttraumatic stress symptoms (PTSS) in a sample of 2,091 Chinese adults 1 month after the COVID-19 outbreak, and found that 4.6% of the participants reported PTS. In another study, Liu et al. (2020) explored the prevalence of PTSS a month after the COVID-19 outbreak in the Chinese areas that have been more affected by the COVID-19. According to their results, 7% of the participants suffered from PTSS. In both studies, the negative psychological symptoms were more prevalent for women.

Given the high amount of people infected in Spain, the escalating number of deaths, and the severe restrictions taken by the Spanish government to ameliorate the virus spread, especially the quarantine, it is quite likely that Spanish mental health is being diminished. To the best of our knowledge, the psychological impact and mental health of the general population living in Spain during the COVID-19 pandemic is unknown. We believe there is an urgent need to deepen our knowledge about Spaniards mental health as a first step to develop psychological interventions, so that the lasting psychological negative consequences of the pandemic can be reduced. We have two main aims. The first one is to explore the mental health status of the general adult population in Spain during the first stages of the COVID-19 outbreak, in terms of psychological impact caused by the pandemic (including intrusion, avoidance, and hyperarousal symptoms), anxiety, depression, and stress. The second one is to examine the extent to which the following variables are associated to psychological impact, anxiety, depression, and stress: (1) demographic variables (e.g., age, gender, monthly family income); (2) degree of concern about the current COVID-19 pandemic; (3) environmental conditions during the home confinement (e.g., number of cohabitants, size of the house); (4) work-related variables (e.g., employment status); (5) changes in daily life as a consequence of the pandemic (e.g., whether the way of working or studying has changed significantly); (6) contact with the COVID-19 disease (e.g., knowing someone who is infected by coronavirus); (7) actual severity of the crisis (number of cases and deaths in Spain) and perceived severity of the crisis; (8) information-related variables (e.g., time spent reading/watching information about coronavirus in the last 24 h); (9) perceived health status (e.g., symptoms experienced in the last 14 days); and (10) leisure activities in which the participant has engaged in the last 24 h.

## Materials and Methods

### Participants

Inclusion criteria were living in Spain during the current COVID-19 crisis and being at least 18 years old. Exclusion criteria were not understanding Spanish well enough to complete the questionnaires. These were stated in the informed consent that was presented before the questionnaire.

A total of 3,055 adults from all Spanish provinces (50) filled the questionnaires. Over half the respondents (*N* = 1,683; 55%) submitted the questionnaires on the first day of data collection, in line with previous similar studies (Wang et al., 2020). Sociodemographic characteristics are comprehensively presented in Table 2. Most respondents were women (75.1%), young adults (age *M* = 32.15 years, *SD* = 12.95; range 18–88), married or cohabiting with partner (38%) or single (34.5%), without children (74.1%), living in a 80–120m^2^ residence (38.4%) with an open air space such as a patio or balcony (64.4%), with a household size of 2–4 people (78.6%), employed or self-employed (53.2%), and well educated (72% hold at least a University degree).

Most participants lived in the provinces of Madrid (52.3%), Barcelona (5.5%), Vizcaya (4%), Guadalajara (3.6%), and Valencia (3.3%). Participants had been born mostly in Spain (94.4%), with the rest indicating 34 different countries of birth — Romania (0.7%), Argentina (0.7%), Colombia (0.5%), Venezuela (0.4%), and France (0.4%) were the most prevalent.

### Instruments

#### Demographics

Participants provided information regarding their gender, age, birth country, Spanish province of residence, marital status, number of children, education level, and monthly family income.

#### Impact of Event Scale-Revised (IES-R; Weiss and Marmar, 1996; Weiss, 2007)

The IES-R is a 22-item self-administered questionnaire designed to measure the magnitude of symptomatic response in the past 7 days to a specific traumatic life event. The response format is a 5-point scale ranging from 0 (not at all or hardly ever) to 4 (a great deal). It is a revised version of the older version, the IES (Horowitz et al., 1979), which included 15 items and two subscales: avoidance and intrusion. The IES-R includes three subscales that measure the three main symptoms of Posttraumatic Stress Disorder (PTSD): avoidance (items 5, 7, 8, 11, 12, 13, 17, and 22), intrusion (items 1, 2, 3, 6, 9, 16, and 20) and hyperarousal (items 4, 10, 14, 15, 18, 19, and 21). A total score can also be obtained as a result of the sum of the scores of the three subscales. To make our data comparable to those by the study conducted by Wang et al. (2020) in Chinese population, the total IES-R score was divided into 0–23 (normal), 24–32 (mild psychological impact), 33–36 (moderate psychological impact), and >37 (severe psychological impact). The IES-R has been validated in the Spanish general population by Baguena et al. (2001), and also in Spanish cancer patients (Gil-Moncayo and Costa-Requena, 2007) showing adequate psychometric properties. In the present study, the instructions and the items were adapted to refer to the current COVID-19 sanitary crisis. The internal consistency of the scores was good for the three subscales (avoidance: α = 0.88; intrusion: α = 0.88; hyperarousal: α = 0.87) and for the total scale (α = 0.95).

#### Depression, Anxiety, and Stress Scales (DASS-21; Lovibond and Lovibond, 1995)

The DASS-21 is a 21-item self-report instrument for mental health assessment consisting of three 7-item subscales: depression (items 3, 5, 10, 13, 16, 17, and 21), anxiety (items 2, 4, 7, 9, 15, 19, and 20), and stress (1, 6, 8, 11, 12, 14, and 18). Participants indicate on a 4-point Likert scale ranging from 0 (Did not apply to me at all) to 3 (Applied to me very much, or most of the time) the degree in which a symptom has been present over the past week. Scores for each subscale were computed by summing the item responses and doubling the result up (Lovibond and Lovibond, 1995; Le et al., 2019) to make them comparable to similar COVID-19 research (Wang et al., 2020). The subscales scores can be allocated on one of 5 levels of severity, as described in Wang et al. (2020) – for depression, normal (0–9), mild (10–12), moderate (13–20), severe (21–27), and extremely severe (28–42); for anxiety, normal (0–6), mild (7–9), moderate (10–14), severe (15–19), and extremely severe (20–42); and for stress, normal (0–10), mild (11–18), moderate (19–26), severe (27–34), and extremely severe (35–42). The DASS-21 has been demonstrated to be a reliable and valid measure in Spanish populations (Bados et al., 2005) and has been previously used in SARS-related research (McAlonan, 2007; Wang et al., 2020). The internal consistency of the scores in the current sample was good for the three subscales (depression: α = 0.89; anxiety: α = 0.86; stress: α = 0.88) and the general distress scale (α = 0.94).

#### Degree of Concern

Participants were asked about the degree to which they were concerned (not at all concerned or very little, somewhat concerned, rather concerned, very concerned) about the following: (1) Health care workers not having the capacity to diagnose and treat the coronavirus; (2) A loved one being infected by coronavirus, (3) Food or health products (such as masks or gloves) shortage, (4) The measures taken by the Government to control the pandemic not being enough, (5) The economic impact of the pandemic, (6) The situation of collective nervousness, (7) Not knowing when this crisis is going to end, and (8) Their psychological state during the crisis.

#### Living and Environmental Conditions During the Home Confinement

Participants provided information about how many people were living together, dependent cohabitants during the home confinement (including children and their ages, as well as other dependents), size of the residence (in squared meters), whether the residence had any open air area (such as a patio or a terrace), the average number of hours a day spent at home during the last week, and the number of days spent without leaving their home.

#### Work-Related Information

Participants provided information regarding their work status, significant modifications in the development of their daily work due to the COVID-19 pandemic, whether they were working during the crisis (options: yes, I’m attending to my workplace, yes, I’m teleworking; no, I stopped working as a consequence of the coronavirus crisis; no; I lost my job because of the coronavirus crisis; no, I didn’t work before the crisis started), whether they thought that there were at risk of losing their jobs as a consequence of the pandemic, and whether they thought that their income was likely to decrease due to the pandemic.

#### Significant Changes in Daily Life

Participants indicated their perceived degree to which the current crisis was affecting their daily life, whether they had had to cancel important activities, whether they had had substantial modifications in the working/studying method, and whether they had to cancel/postpone any travels.

#### Contact With the COVID-19 Disease

Participants indicated whether they knew someone infected by coronavirus, had had close contact with someone then diagnosed with coronavirus in the previous 14 days, had had close contact with someone who had coronavirus symptoms in the previous 14 days, had had symptoms of coronavirus themselves, had taken the coronavirus test with a negative result, had taken the coronavirus test with a positive result, or had experienced nothing of the above.

#### Actual and Perceived Severity of the Situation

Respondents indicated the degree to which they perceived the current crisis as severe (0–10) and we also collected the official number of people infected by coronavirus and of deaths by coronavirus in Spain the day that they filled the questionnaires (these data were obtained from the WHO website, World Health Organization, 2020).

### Information About the COVID-19 Pandemic

Participants indicated the main information media they had used to receive information about the COVID-19 crisis, their need for more information, and the number of hours invested in the previous 24 h in watching/reading information about the coronavirus crisis.

#### Health Status

Participants indicated their perceived health level (from 0 = very bad, to 10 = excellent), their perception of belonging to the high-risk population in case of being infected by coronavirus, the symptoms they had experienced in the previous 14 days (fever of at least 38°C, sore throat, headaches, muscle or joint pain, cough, respiratory distress, fatigue, none of the above), and their utilization of any health services related to coronavirus during the last 14 days.

#### Leisure Activities During Home Confinement

Participants provided information regarding whether they had carried out the following activities in the previous 24 h: physical exercise, watching films/series, reading, watching TV, making crafts or any artistic activity, playing, browsing or sharing contents in social networks, talking to someone (face to face or via telephone, instant messaging, videocalls…), other leisure activities, or none of the above.

### Procedure

The study was approved by the ethics committees at the first and second authors’ universities. Given the restrictions imposed over the face-to face interaction during the data-collection period, data were collected online, through a Google Forms questionnaire. Data collection period comprised between the 17th of March 2020 (2 days after the state of alarm was implemented in Spain and a week after the WHO declared the outbreak a pandemic) and the 24th of March 2020. Participants were contacted by email and social networks (Facebook, Instagram, Twitter, LinkedIn, and WhatsApp), following a snowball approach. All respondents provided informed consent prior accessing the questionnaires.

### Data Analyses

Descriptive statistics were computed for the sociodemographic characteristics of the sample and the study variables, consisting of frequencies and percentages for categorical variables and means and standard deviations (*SD*) for scale and ordinal variables. For the mental health variables, skewness and kurtosis values were obtained (see Table 1) with no further interpretation due to the large sample size (Field, 2009; Ghasemi and Zahediasl, 2012).

**TABLE 1:** Means, standard deviations, Skewness and Kurtosis for Impact of event, stress, anxiety, and depression.

	Mean	*SD*	Skewness		Kurtosis	
				
			Value	*SE*	Value	*SE*
Impact of event	27.95	19.21	0.66	0.04	−0.35	0.09
Stress	11.04	10.04	0.83	0.04	−0.15	0.09
Anxiety	6.23	8.19	1.71	0.04	2.68	0.09
Depression	9.88	9.98	1.18	0.04	0.66	0.09

*SD, standard deviation. SE, standard error.*

**TABLE 2:** Association between demographic variables and the psychological impact of the COVID-19 pandemic as well as mental health status during the pandemic (*N* = 3055).

Variables	*N* (%)	Impact of event				Stress				Anxiety				Depression			
					
		*M* (*SD*)	*t*/F*	*p*	*g*/η^2^*	*M* (*SD*)	*t*/F*	*p*	*g*/η^2^*	*M* (*SD*)	*t*/F*	*p*	*g*/η^2^*	*M*(*SD*)	*t*/F*	*p*	*g*/η^2^*
Gender**																	
Male	744 (24.4)	21.35 (17.04)	11.75^1^	<0.001	0.46	8.01 (8.63)	10.47^1^	<0.001	0.41	4.12 (6.52)	9.39 ^1^	<0.001	0.34	7.71 (8.91)	7.30 ^1^	<0.001	0.29
Female	2293 (75.1)	30.10 (19.42)				12.02 (10.27)				6.92 (8.57)				10.56 (10.18)			
Other	18 (0.6)	26.83 (16.46)				11.67 (8.97)				4.78 (6.86)				13.56 (12.15)			
**Country of origin**																	
Spain	2283 (94.4)	27.90 (19.28)	−0.60	0.55	0.05	11.02 (10.07)	−0.49	0.62	0.04	6.16 (8.19)	−1.74	0.08	0.14	9.80 (9.98)	−1.88	0.06	0.15
Other	172 (5.6)	28.80 (18.17)				11.41 (9.40)				7.28 (8.23)				11.27 (9.79)			
**Region**																	
Madrid	1598 (52.3)	27.22 (18.75)	−2.18^1^	0.03	0.08	11.04 (9.81)	−0.02	0.98	0	6.05 (8.02)	−1.25	0.21	0.05	9.52 (9.73)	−2.06 ^1^	0.04	0.08
Other	1457 (47.7)	28.75 (19.69)				11.04 (10.28)				6.42 (8.38)				10.27 (10.23)			
**Marital status**																	
Married/cohabiting with a partner	1160 (38)	24.72 (18.15)^a^	27.61^1^	<0.001	0.04	9.23 (9.13)^a^	31.59^1^	<0.001	0.04	5.07 (7.32)^a^	19.47^1^	<0.001	0.03	7.20 (8.54)^a^	44.07^1^	<0.001	0.06
In a relationship but not cohabiting	748 (24.5)	33.26 (20.74)^b^				13.74 (10.99)^b^				7.97 (9.35)^b^				12.51 (10.94)^b^			
Single	1054 (34.5)	28.42 (18.55)^c^				11.59 (9.99)^c^				6.56 (8.14)^c^				11.26 (10.14)^b^			
Separated/divorced	76 (2.5)	19.25 (15.33)^d^				5.89 (6.53)^d^				2.47 (4.51)^d^				6.86 (7.89)^a^			
Widow(er)	17 (0.6)	24,53 (17.76)^abcd^				4.71 (5.05)^d^				4 (9.22)^abcd^				4.94 (5.25)^a^			
**N° of children**																	
No children	2265 (74.1)	29.21 (19.35)^a^	12.86	<0.001	0.01	11.80 (10.23)^a^	17.11^1^	<0.001	0.02	6.75 (8.38)^a^	12.06^1^	<0.001	0.01	10.89 (10.29)^a^	31.24^1^	<0.001	0.03
One	294 (9.6)	24.35 (19.35)^b^				8.95 (9.60)^b^				4.74 (7.43)^b^				7.62 (8.91)^b^			
Two	388 (12.7)	24.54 (17.99)^b^				8.80 (8.91)^b^				4.60 (7.25)^b^				6.51 (8.12)^b^			
Three or more	108 (3.6)	23.50 (17.03)^b^				8.80 (8.61)^b^				5.17 (8.15)^ab^				7.06 (7.89)^b^			
**Education level**																	
No studies	3	52.33 (7.09)^df^	4.92^1^	<0.001	0.01	28 (10)^ab^	4.37^1^	<0.001	0.01	11.33 (17.93)^abc^	4.46^1^	<0.001	0.01	27.33 (12.86)^abc^	10.92^1^	<0.001	0.02
Primary education	29	24.24 (19.56)^eg^				7.93 (9.08)^ab^				6 (8.78)^abc^				8.56 (9.83)^abc^			
Secondary compulsory education	91 (3)	32.01 (20.78)^bcde^				11.98 (10.31)^ab^				9.30 (9.19)^a^				12.41 (11.51)^c^			
Secondary post-compulsory education	294 (9.6)	28.50 (19.60)^bcde^				11,13 (10.35)^ab^				6.49 (9.10)^ab^				11.66 (11.22)^c^			
Professional training	439 (14.4)	27.47 (20.14)^bcde^				10.55 (10.33)^ab^				6.22 (8.22)^ab^				9.87 (10.51)^bc^			
University degree	1435 (47)	29.12 (19.47)^bde^				11.68 (10.30)^a^				6.49 (8.23)^ab^				10.52 (10.05)^c^			
Master’s degree	610 (20)	26.13 (17.59)^acde^				10.38 (9.23)^ab^				5.56 (7.63)^bc^				8.15 (8.53)^b^			
Ph.D	154 (5)	22.40 (16.76)^afg^				8.66 (8.20)^b^				4.01 (6.35)^c^				5.80 (6.88)^a^			
Montly income***																	
<1000 €	293 (9.6)	30.41 (19.74)^bc^	5.97^1^	<0.001	0.01	12.78 (10.77)^a^	3.58^1^	<0.01	0.01	7.80 (8.80)^b^	6.63^1^	<0.001	0.01	12.81 (11.26)^a^	10.27^1^	<0.001	0.02
1000 – 1500 €	496 (16.2)	29.83 (20.51)^bc^				11.52 (10.42)^ab^				7.12 (8.98)^bd^				11.25 (10.57)^ab^			
1500 – 2000 €	524 (17.2)	30.35 (19.55)^b^				11.71 (9.98)^ab^				6.99 (8.56)^bcd^				10.36 (10.02)^bc^			
2000 – 2500 €	491 (16.1)	26.37 (17.92)^ac^				10.33 (9.98)^b^				5.63 (7.55)^ad^				9.36 (9.84)^bcd^			
2500 – 3000 €	380 (12.4)	27.54 (19.07)^abc^				10.75 (9.99)^ab^				5.95 (8.31)^ab^				9.02 (9.33)^cd^			
3000 – 3500 €	302 (9.9)	26.26 (18.75)^ac^				10.36 (9.24)^ab^				5.36 (7.51)^ac^				8.65 (8.97)^cd^			
>3500 €	541 (17.7)	25.10 (18.24)^a^				10.08 (9.68)^b^				5.01 (7.33)^a^				8.18 (9.13)^d^			
**Age groups**																	
18 – 24	1201 (39.3)	32.06 (19.76)^a^	29.64^1^	<0.001	0.05	13.18 (10.64)^a^	33.60^1^	<0.001	0.05	7.49 (8.81)^a^	18.18^1^	<0.001	0.03	12.80 (10.70)^a^	48.28^1^	<0.001	0.07
25 – 34	795 (26)	28.38 (19.22)^b^				11.52 (10.02)^b^				6.66 (8.38)^a^				9.81 (9.83)^b^			
35 – 44	476 (15.6)	23.90 (18.02)^c^				9.21 (8.89)^c^				5.06 (7.09)^b^				7.02 (8.10)^c^			
45 – 54	334 (10.9)	23.63 (16.74)^c^				8.38 (8.51)^c^				4.38 (6.99)^bc^				6.99 (8.46)^c^			
55 – 64	188 (6.2)	22.36 (17.25)^c^				7.15 (8.28)^c^				3.85 (6.67)^bc^				5.82 (7.67)^cd^			
>65	61 (2)	13.95 (12.90)^d^				3.38 (4.85)^d^				2.13 (5.43)^c^				3.80 (5.29)^d^			
	*M*(*SD*)	Pearson’s *r*		*p*		Pearson’s *r*		*p*		Pearson’s *r*		*p*		Pearson’s *r*		*p*	
**Age**	32.14 (12.96)	−0.206		<0.001		−0.226		<0.001		−0.171		<0.001		−0.245		<0.001	

*Differences in mean level between categories of dichotomous variables were assessed via *t*-test and Hedges’ *g* effect size statistic was obtained (interpretation: negligible < 0.20 < small < 0.50 < medium < 0.80 < large). For multiple-category variables, one-way ANOVAs were used, and categories with a different superscript letter show a significant difference between them in the psychological impact variable mean. In these cases, the effect size was assessed via η^*2*^ (interpretation: negligible < 0.01 < small < 0.06 < medium < 0.14 < large). **For gender comparison, given the low number of people who responded “other” (*N* = 18) only men and women have been included. The means are included for the three categories (men, women, and other), but mean differences were only tested between women and men via *t*-test. *** For monthly income 28 persons selected the option “rather not to say.” These analyses have been conducted with the participants who indicated their family monthly income. ^*1*^Homoscedascity could not be assumed for these variables and thus the *t*-test results adjusted for non-homogeneous variances were used, and in the case of ANOVAS, *post hoc* Games-Howell tests were used.

Parametric tests were then carried out, since again the large sample size (>30) allows for the shape of the data to not cause problems in these instances (Ghasemi and Zahediasl, 2012; Kwak and Kim, 2017). Differences in mean level of mental health variables between categories of dichotomous variables (e.g., perceived risk of losing job) were assessed via *t*-test, taking the test results adjusted for non-homogeneous variances when necessary if a significant Levene’s test was found. For multiple-category variables (e.g., gender group, age groups, education, marital status, impact on work), one-way ANOVA was used, with *post hoc* Tukey (for homogeneous variances) or Games-Howell (for non-homogeneous variances) between group comparisons in case of a significant overall *F*-value. Appropriate effect size statistics that adjust for differences in group sizes were obtained — Hedges’ *g* for *t*-tests and η^2^ for ANOVAs. Bivariate associations between mental health variables (psychological impact, anxiety, stress and depression) and age (continuous variable) were assessed via Pearson’s correlation coefficient *r*. Spearman’s correlation coefficient ρ was used to test bivariate associations between mental health variables and ordinal variables (i.e., degree of concern, perceived severity, perceived health).

All tests were two-tailed, with a significance level of *p* < 0.05. Statistical analyses were performed using SPSS Statistics 25.0.

## Results

### Psychological Impact and Mental Health

The psychological impact of COVID-19 pandemic in Spain, measured through the IES-R scale, revealed a sample mean score of 27.94 (*SD* = 19.21; see Table 1). A total of 930 respondents (30.4%) showed severe psychological impact (score > 37), 188 (6.2%) reported a moderate psychological impact (score 33-36); 441 (14.4%) scored in the range for mild psychological impact (score 24–32), and 1,496 participants (49%) reported minimal psychological impact (score < 23).

The global mental health, as assessed by the DASS-21 scale showed a mean score of 27.14 (*SD* = 25.54). Regarding the DASS-21 subscales (see Table 1), the mean score for anxiety was 6.23 (*SD* = 8.19). A total of 2,118 respondents (69.3%) were considered to have normal levels of anxiety (score 0–6), 162 participants (5.3%) showed mild anxiety (score 7–9), 346 (11.3%) showed moderate anxiety (score 10–14), 147 (4.8%) showed severe anxiety (score 15–19) and 282 (9.2%) showed extremely severe anxiety (score > 20). With regards to depression, the mean score was 9.88 (*SD* = 9.98). A total of 1,805 respondents (59.1%) showed normal levels (score 0–9), 347 (11.4%) mild depression (score 10–12), 451 (14.8%) moderate depression (score 13–20), 191 (6.3%) severe depression (score 21–27) and 261 (8.5%) extremely severe depression (score > 28). Finally, the mean for stress was 11.04 (*SD* = 10.04). Of participants, 1,772 (58%) showed normal stress levels (score 0–10), 599 (19.6%) mild stress (score 11–18), 408 (13.4%) moderate stress (score 19–26), 22 (6.9%) severe stress (27–34), and 64 (2.1%) extremely severe stress (>35).

### Demographics

Table 2 shows the descriptive data for all demographic variables as well as the associations between such variables and psychological impact, stress, anxiety and depression. Women showed significantly higher levels in all the variables. The mean age was 32.14 (*SD* = 12.96). Over 65% of the participants were 34 years old or younger. Pearson’s correlational analyses revealed that the psychological impact of the COVID-19 crisis seems to ameliorate as people get older. Thus, participants aged 18–24 showed the highest psychological impact, followed by the group aged 25–34. No differences were found in the psychological impact reported by people who were between 35 and 64 years old. Participants who were 65 years old or over showed the lowest psychological impact. Participants living in Spanish regions different from Madrid reported higher levels of psychological impact and depression (even though the situation, in terms of infected people and deaths caused by COVID-19, was more severe in Madrid). As for marital status, mean differences were significant for all the study variables. The *post hoc* tests for psychological impact showed that mean differences were significant between those who were in a relationship but not cohabiting (who showed the highest psychological impact) and those who were married, separated, and single. Married participants and those cohabiting with their partner showed significantly lower psychological impact than single participants, while separated participants showed significantly lower distress than those who were single or married. As for stress, the *post hoc* test showed that mean differences between all groups (but widowed participants, probably due to the small sample size of this group) were significant. The most stressed, anxious, and depressed participants were those who were in a relationship but not cohabiting, followed by single participants. Those who had children showed lower psychological impact, stress, anxiety, and depression than those with no children. *Post hoc* tests revealed no differences between participants with one, two or three, or more children. Considering educational level, participants with a Ph.D. showed lower psychological impact, stress, anxiety, and depression than those with high school studies, professional training, and university undergraduate studies. Participants with high family monthly income (3,000–3,500 €/month) showed lower psychological impact than those whose family income was lower than 2,000 €/month. All the effect sizes were small (see Table 2), except for differences in depression by marital status and by age groups, which were medium.

### Degree of Concern

Table 3 shows the mean scores in each of the concerns about the COVID-19 that were included in the study, as well as the Spearman’s correlations between each concern and psychological impact of the event, stress, anxiety, and depression. The average level of concern (mean of all the items) was 2.93 (*SD* = 0.55; range 1–4). What worried participants the most was the economic impact of the pandemic (*M* = 3.37; *SD* = 0.72), a loved one being infected by coronavirus (*M* = 3.35; *SD* = 0.75) and not knowing when this health crisis is going to end (*M* = 3.05; *SD* = 0.84). All the concerns showed positive and significant associations with psychological impact, stress, anxiety and depression. The concern that was more strongly associated with distress was “My psychological state during the crisis” followed by “Not knowing when this crisis is going to end.”

**TABLE 3:** Association between types and degree of concern about the COVID-19 pandemic and the psychological impact of the COVID-19 pandemic as well as mental health status during the pandemic (*N* = 3055).

Concerns	*M* (*SD*)	Impact of event	Stress	Anxiety	Depression
Health care workers not having the capacity to diagnose and treat the coronavirus (1–4)	2.97 (0.99)	0.19***	0.10***	0.12***	0.09***
A loved one being infected by coronavirus (1–4)	3.35 (.75)	0.28***	0.17***	0.19***	0.13***
Food or health products (such as masks or gloves) shortage (1–4)	2.54 (1)	0.24***	0.14***	0.17***	0.11***
The measures taken by the Government to control the pandemic not being enough (1–4)	2.93 (0.90)	0.28***	0.16***	0.17***	0.13***
The economic impact of the pandemic (1–4)	3.37 (0.72)	0.20***	0.11***	0.09***	0.09***
The situation of collective nervousness (1–4)	2.95 (0.87)	0.30***	0.22***	0.20***	0.17***
Not knowing when this crisis is going to end (1–4)	3.05 (0.84)	0.41***	0.30***	0.27***	0.29***
My psychological state during the crisis (1–4)	2.37 (1.01)	0.59***	0.55***	0.48***	0.54***
*Average level of concern (1–4)*	*2.93 (0.55)*	0.49***	0.35***	0.35***	0.31***

Spearman’s rho correlation coefficient (ρ) was used for correlation calculations between concerns and mental health variables. *** *p* < 0.001.

### Living and Environmental Conditions During the Home Confinement

Most of the participants had a household of two people (24.5), three people (25.9) or four people (28.2), and 10.1% lived alone. Differences in distress by household size were significant for psychological impact, stress and depression (see Table 4) with small effect sizes. The *post-hoc* tests for psychological impact revealed that differences were significant only between individuals with a household of one/two (who suffered the lower psychological impact) and participants with a household of four people. As for stress, differences were significant between participants with a household of one or two people (the less stressed) and participants with a household of three or four people (the most stressed). The lower psychological impact was found in participants with a household of two, and their depression levels were significantly lower than of those with a household of three or four, who showed the highest depression levels.

**TABLE 4:** Association between living and environmental conditions during the home confinement and the psychological impact of the COVID-19 pandemic as well as mental health status during the pandemic (*N* = 3055).

Variables	*N* (%)	Impact of event				Stress				Anxiety				Depression			
					
		*M* (*SD*)	*t*/F*	*p*	*g*/η^2^*	*M* (*SD*)	*t*/F*	*p*	*g*/η^2^*	*M* (*SD*)	*t*/F*	*p*	*g*/η^2^*	*M*(*SD*)	*t*/F*	*p*	*g*/η^2^*
**Household size**																	
One person	308 (10.1)	26.05 (18.43)^a^	4.25	<0.01	0.01	9.50 (9.85)^a^	5.78^1^	<0.001	0.01	6.02 (8.07)	1.43 ^1^	0.21	0	9.36 (9.80)^ab^	3.95^1^	<0.01	0.01
Two people	750 (24.5)	25.88 (18.72)^a^				9.88 (9.37)^a^				5.71 (7.98)				8.67 (9.42)^a^			
Three people	790 (25.9)	28.29 (19.40)^ab^				11.49 (10.31)^b^				6.29 (8.45)				10.51 (10.21)^b^			
Four people	863 (28.2)	29.89 (19.55)^b^				12.05 (10.27)^b^				6.76 (8.40)				10.60 (10.35)^b^			
Five people	257 (8.4)	28.72 (19.54)^ab^				11.14 (9.90)^ab^				6.04 (7.63)				9.77 (9.78)^ab^			
6 or more	87 (2.8)	27.91 (18.33)^ab^				12.16 (10.31)^ab^				5.95 (7.49)				9.68 (8.43)^ab^			
**Living with kids**																	
*Don’t have kids (Reference)*	*2129 (69.69)*	*28.99 (19.26)*				*11.73 (10.24)*				*6.63 (8.24)*				*10.85 (10.26)*			
Have kids but not living with me	118 (3.86)	19.99 (17.35)	−5.63	<0.001	0.47	5.64 (7.86)	−8.42	<0.001	0.60	3.10 (5.91)	−6.48	<0.001	0.44	6.00 (7.29)	−7.22	<0.001	0.48
At least one kid < 5 years	212 (6.94)	27.15 (19.18)	−1.40	0.16	0.10	11.10 (10.07)	−0.91	0.37	0.06	6.24 (8.46)	−0.66	0.51	0.05	8.04 (9.12)	−4.49	<0.001	0.28
At least one kid 6–10 years	153 (5.01)	25.80 (18.37)	−2.15	0.03	0.17	10.34 (9.34)	−1.84	0.07	0.14	4.73 (7.01)	−3.35	<0.01	0.23	7.01 (8.10)	−5.87	<0.001	0.38
At least one kid 11–15 years	181 (5.92)	24.54 (18.01)	−3.32	<0.01	0.23	8.72 (8.92)	−4.54	<0.001	0.30	4.61 (7.77)	−3.50	<0.01	0.25	7.52 (9.33)	−4.80	<0.001	0.33
At least one kid 16–20 years	181 (5.92)	24.85 (18.98)	−2.94	<0.01	0.22	8.76 (9.27)	−4.31	<0.001	0.29	5.54 (8.44)	−1.74	0.08	0.13	7.31 (8.80)	−5.40	<0.001	0.35
**Living with elderly**																	
No	2824 (92.44)	27.77 (19.11)	−1.84	0.07	0.13	10.99 (10.00)	−0.84	0.40	0.06	6.14 (8.07)	−1.78^1^	0.08	0.14	9.83 (9.91)	−1.01	0.31	0.07
Yes	231 (7.56)	30.19 (20.31)				11.58 (10.42)				7.29 (9.59)				10.52 (10.68)			
***M*^2^ residence**																	
<50	194 (6.4)	28.02 (19.27)^ab^	2.90	0.03	0	11.03 (10.28)^ab^	3.44	0.02	0	6.65 (8.40)^ab^	4.05^1^	<0.01	0	10.54 (9.86)^ab^	3.23^1^	0.02	0
50–80	925 (30.3)	28.94 (19.48)^a^				11.36 (10.12)^a^				6.65 (8.56)^a^				10.23 (10.42)^a^			
80–120	1173 (38.4)	28.26 (19.14)^ab^				11.43 (10.17)^a^				6.39 (8.19)^a^				10.12 (9.91)^a^			
>120	763 (25)	26.26 (19.91)^b^				10.04 (9.61)^b^				5.35 (7.63)^b^				8.92 (9.52)^b^			
**Overcrowding index**																	
<1.17	1780 (58.27)	26.60 (18.96)	4.57^1^	<0.001	0.17	10.41 (9.81)	4.06^1^	<0.001	0.15	5.92 (8.18)	2.46^1^	0.01	0.09	9.40 (9.79)	3.14^1^	<0.01	0.12
≥1.17	1275 (41.73)	29.83 (19.41)				11.92 (10.23)				6.66 (8.20)				10.55 (10.20)			
**Open-air space**																	
Yes	1966 (64.4)	27.33 (19.18)	2.39	0.02	0.09	10.86 (10.10)	1.37	0.17	0.05	6.06 (8.15)	1.63	0.10	0.06	9.89 (10.04)	−0.07	0.95	0
No	1089 (35.6)	29.07 (19.24)				11.37 (9.91)				6.55 (8.28)				9.86 (9.87)			
**Days without leaving residence**																	
0	660 (21.6)	26.08 19.33)^a^	5.20 ^1^	<0.001	0.01	10.24 (10.16)^a^	4.20^1^	<0.01	0.01	5.60 (7.88)^a^	4.52^1^	<0.001	0.01	8.57 (9.34)^a^	7.16^1^	<0.001	0.01
1	380 (12.4)	26.94 (18.24)^a^				10.71 (9.35)^ab^				5.67 (7.63)^a^				8.91 (8.80)^ac^			
2	283 (9.3)	27.83 (16.92)^ab^				10.96 (9.01)^ab^				5.59 (7.08)^a^				9.39 (9.03)^abc^			
3	373 (12.2)	26.28 (19.32)^a^				9.97 (9.26)^a^				5.81 (7.68)^a^				9.42 (9.80)^ac^			
4	452 (14.8)	27.92 (18.60)^ab^				11.10 (9.77)^ab^				6.29 (8.28)^a^				10.59 (10.19)^bc^			
5 or more	907 (29.7)	30.48 (20.21)^b^				12.20 (10.84)^b^				7.25 (9.02)^b^				11.23 (10.93)^b^			
**Daily h. at home**																	
<10	66 (2.2)	27.95 (20.80)	0.09	0.96	0	12.64 (11.67)	0.64	0.59	0	8.03 (9.27)	1.26	0.29	0	10.15 (10.58)	1.91	0.13	0
10–14	212 (6.9)	28.05 (19.74)				10.80 (10.44)				6.17 (8.34)				8.99 (9.30)			
15–19	425 (13.9)	27.49 (19.21)				11.17 (10.09)				6.44 (8.08)				9.06 (9.42)			
20–24	2352 (77)	28.02 (19.13)				10.99 (9.94)				6.14 (8.17)				10.10 (10.11)			

* Differences in mean level between categories of dichotomous variables were assessed via *t*-test and Hedges’ *g* effect size statistic was obtained (interpretation: negligible < 0.20 < small < 0.50 < medium < 0.80 < large). For multiple-category variables, one-way ANOVAs were used, and categories with a different superscript letter show a significant difference between them in the psychological impact variable mean. In these cases, the effect size was assessed via η^2^ (interpretation: negligible < 0.01 < small < 0.06 < medium < 0.14 < large). ^1^Homoscedascity could not be assumed for these variables and thus the *t*-test results adjusted for non-homogeneous variances were used, and in the case of ANOVAS, *post hoc* Games-Howell tests were used.

Participants who did not have kids were compared to those who had kids but not living with them, those living with kids aged less than 5 years old, 6–10 years old, 11–15 years old, and older than 16 years old. Participants with children aged less than 5 years old showed equivalent levels of psychological impact and anxiety than those with no children. However, participants with children who were older than 10 years old and those who had children but did not live with them showed lower psychological impact and anxiety than those without children. Participants with children aged <5 years old and with children between 6 to 10 years old were equally stressed as participants with no children. However, participants with children older than 11 years old and those who have kids that did not live with them showed lower stress levels. With regards to depression levels, participants with children (irrespective of their age) showed lower levels of depression than participants with no children. Participants living with elderly family members did not show differences in their levels of distress compared to those not living with elderly people. Effect sizes were small, expect for the effect size of having kids who lived elsewhere on stress, which was medium.

The size of the residence was associated with the respondents’ mental health. Participant with houses sized more than 120 square meters showed lower psychological impact, stress, anxiety and depression. In order to explore whether there was an association between the resident density of the house and the levels of distress, an overcrowding index was calculated based on the residence size and the household size. Participants living with a low overcrowding index showed lower distress than those with a high overcrowding index. Respondents whose residence had an open-air space showed slightly lower psychological impact. At the time of data collection, almost 30% of the participants had been confined at home for more than 5 days. The more days without leaving their home, the higher the distress levels. In the week prior participating in the study (i.e., before the state of alarm, but after the start of the outbreak in Spain), 77% of the respondents had spent an average of 20–24 h at home. No significant associations were found in terms of hours spent at home the week before. All effect sized were small.

### Work-Related Information

Descriptive data for work-related variables as well as their association with psychological impact, stress, anxiety, and depression are included in Table 5. Many participants (35%) were teleworking, while 15% had temporarily stopped working due to the crisis, 3.2% lost their jobs due to the crisis and 10.4% continued working on site. More than 36% of the participants perceived a risk of losing their jobs and more than 44% a risk of decreased income because of the pandemic. Differences were significant for all the work-related variables included. *Post hoc* tests showed that participants with the highest psychological impact, stress, and anxiety levels were students, while those least affected were retired. There were no differences between self-employed, employed, and unemployed participants for psychological impact, stress, and anxiety. For depression, students and unemployed respondents showed higher levels than the rest of participants, while retired individuals showed lower depression symptoms than the rest of groups. With regards to the work situation during the crisis, participants who were working on site, those who had lost their jobs due to the crisis, and those who had stopped working due to the crisis showed higher levels of psychological impact, stress, anxiety, and depression than respondents who were teleworking. Finally, participants with higher perceived levels of losing their jobs during the COVID-19 crisis (considering only the participants who were working before the crisis started) and those with higher perceived risk of reduced family income as a consequence of the crisis reported significantly higher scores in all the variables included. All the effect sizes were small (see Table 5).

**TABLE 5:** Association between work-related variables and the psychological impact of the COVID-19 pandemic as well as mental health status during the pandemic (*N* = 3055).

Variables	*N* (%)	Impact of event				Stress				Anxiety				Depression			
					
		*M* (*SD*)	*t*/F*	*p*	*g*/η^2^*	*M* (*SD*)	*t*/F*	*p*	*g*/η^2^*	*M* (*SD*)	*t*/F*	*p*	*g*/η^2^*	*M* (*SD*)	*t*/F*	*p*	*g*/η^2^*
**Employment status**																	
Employed	1412 (46.2)	26.16 (18.41)^a^	18.55^1^	<0.001	0.02	10.04 (9.45)^a^	21.87^1^	<0.001	0.03	5.68 (7.71)^a^	9.13^1^	<0.001	0.01	8.24 (8.99)^a^	39.79^1^	<0.001	0.05
Self-employed	215 (7)	26.15 (19.40)^a^				10.25 (10.42)^a^				5.67 (8.51)^ab^				8.05 (9.55)^a^			
Unemployed	258 (8.4)	29.09 (21.12)^ac^				11.02 (10.18)^a^				6.86 (9)^ab^				11.25 (10.97)^b^			
Retired	82 (2.7)	16.95 (14.36)^b^				5.15 (6.64)^b^				2.83 (5.82)^c^				4.59 (6.01)^c^			
Student	1037 (33.9)	31.26 (19.51)^c^				13.06 (10.55)^c^				7.16 (8.59)^b^				12.61 (10.59)^b^			
With invalidity	23 (0.8)	26.17 (19.30)				8 (7.98)				6.87 (8.22)				9.48 (10.72)			
Homemaker	28 (0.9)	32.29 (16.85)				12.64 (9.48)				6.79 (7.80)				9.07 (10.35)			
Working during crisis**																	
Yes, on site	318 (10.4)	30.21 (20.86)^a^	7.81^1^	<0.001	0.01	11.85 (10.77)^a^	5.80^1^	<0.01	0.01	7.18 (8.47)^a^	7.40^1^	<0.001	0.01	8.92 (9.33)^ab^	14.37^1^	<0.001	0.02
Yes, from home	1070 (35)	25.42 (17.99)^b^				9.81 (9.20)^b^				5.19 (7.41)^b^				7.88 (8.78)^a^			
No, I stopped due to the crisis	457 (15)	28.95 (19.33)^a^				11.30 (10.19)^a^				6.59 (8.32)^a^				10.42 (10.14)^bc^			
No, I’ve lost my job due to crisis	98 (3.2)	30.03 (21.32)^ab^				12.29 (10.98)^ab^				7.08 (9.84)^ab^				12.96 (11.66)^c^			
Risk of losing job***																	
No	1115 (36.5)	24.01 (17.99)	−8.27^1^	<0.001	0.42	9.09 (9.12)	−7.06^1^	<0.001	0.36	4.91 (7.32)	−5.91^1^	<0.001	0.30	7.27 (8.49)	−7.35^1^	<0.001	0.38
Yes	657 (21.5)	31.71 (19.46)				12.54 (10.39)				7.29 (8.63)				10.71 (10.08)			
**Risk of decreased income**																	
No	1355 (44.4)	24.31 (17.76)	−9.61^1^	<0.001	0.35	9.23 (9.16)	−9.13^1^	<0.001	0.33	4.97 (7.28)	−7.76^1^	<0.001	0.28	8.13 (8.83)	−8.95^1^	<0.001	0.32
Yes	1700 (55.6)	30.85 (19.83)				12.48 (10.47)				7.22 (8.73)				11.28 (10.60)			

* Differences in mean level between categories of dichotomous variables were assessed via *t*-test and Hedges’ *g* effect size statistic was obtained (interpretation: negligible < 0.20 < small < 0.50 < medium < 0.80 < large). For multiple-category variables, one-way ANOVAs were used, and categories with a different superscript letter show a significant difference between them in the psychological impact variable mean. In these cases, the effect size was assessed via η^2^ (interpretation: negligible < 0.01 < small < 0.06 < medium < 0.14 < large). ** Analyses conducted with the sample of 1943 participants who had a job just before the COVID-19 outbreak. *** Analyses conducted with the sample of 1772 participants who were employed during the COVID-19 outbreak. ^1^Homoscedascity could not be assumed for these variables and thus the *t*-test results adjusted for non-homogeneous variances were used, and in the case of ANOVAS, *post hoc* Games-Howell tests were used.

### Significant Changes in Daily Life

A total of 57% of the participants considered that the COVID-19 crisis had impacted to a great deal or extremely on their daily life (see Table 6). Those respondents who considered that the impact of COVID-19 crisis in their daily life had been high showed increased levels of psychological impact, stress, anxiety, and depression (*post hoc* tests revealed that all the differences were significant except the comparison between the categories “almost none impact” and “a little impact” for the variables stress, anxiety, and depression). More than 84% of the participants indicated that since the COVID-19 crisis started, they had suffered substantial modifications in their work or studies routines, more than 88% had to cancel significant activities, and more than 65% had canceled or postponed any travels. Those who reported significant modifications or cancelation of relevant activities and travels showed worse mental health (in all the variables) that those who did not. The effect sizes for the ANOVA that compared the levels of impact of the event and stress by the degree of perceived impact of the COVID-19 on their daily lives were moderate, while the rest were small.

**TABLE 6:** Association between the presence of significant changes in daily life due to the COVID-19 pandemic and the psychological impact of the COVID-19 pandemic as well as mental health status during the pandemic (*N* = 3055).

Variables	*N* (%)	Impact of event				Stress				Anxiety				Depression			
					
		*M* (*SD*)	*t*/F*	*p*	*g*/η^2^*	*M* (*SD*)	*t*/F*	*p*	*g*/η^2^*	*M* (*SD*)	*t*/F*	*p*	*g*/η^2^*	*M* (*SD*)	*t*/F*	*p*	*g*/η^2^*
**Impact on daily life**																	
Almost none	51 (1.7)	4.86 (7.52)^a^	63.49^1^	<0.001	0.08	4.86 (7.53)^a^	54.24^1^	<0.001	0.07	2.78 (5.08)^a^	31.40^1^	<0.001	0.04	5.22 (7.40)^a^	36.98^1^	<0.001	0.05
A little	370 (12.1)	6.75 (8.27)^b^				6.75 (8.27)^a^				3.86 (5.78)^a^				6.94 (8.28)^a^			
Quite	892 (29.2)	9.57 (8.90)^c^				9.57 (8.90)^b^				5.23 (7.13)^b^				8.48 (8.88)^b^			
A great deal	1078 (35.3)	11.72 (9.93)^d^				11.72 (9.93)^c^				6.41 (8.16)^c^				10.14 (9.92)^c^			
Extremely	664 (21.7)	14.77 (11.14)^e^				14.77 (11.14)^d^				8.83 (10.00)^d^				13.34 (11.37)^d^			
**Work/Studies modifications**																	
No	264 (8.6)	22.38 (16.66)	−5.87^1^	<0.001	0.34	8.65 (9.23)	−4.69	<0.001	0.28	4.83 (7.06)	−3.39^1^	<0.01	0.19	7.96 (9.11)	−3.67^1^	<0.001	0.22
Yes	2571 (84.2)	28.80 (19.35)				11.48 (10.13)				6.40 (8.25)				10.15 (10.01)			
**Cancelation of activities**																	
No	356 (11.7)	23.01 (18.76)	−5.18	<0.001	0.29	8.15 (9.65)	–5.82	<0.001	0.33	4.55 (6.80)	−4.81^1^	<0.001	0.23	7.46 (9)	−5.33^1^	<0.001	0.28
Yes	2699 (88.3)	28.60 (19.18)				11.42 (10.03)				6.45 (8.34)				10.20 (10.06)			
**Cancel/postpone any travels.**																	
No	1051 (34.4)	26.37 (19.13)	−3.29	<0.01	0.13	10.20 (9.97)	−3.35	<0.01	0.13	5.91 (7.74)	−1.58^1^	0.12	0.06	9.53 (10.07)	−1.39	0.17	0.05
Yes	2004 (65.6)	28.78 (19.21)				11.48 (10.05)				6.39 (8.42)				10.06 (9.93)			

* Differences in mean level between categories of dichotomous variables were assessed via *t*-test and Hedges’ *g* effect size statistic was obtained (interpretation: negligible < 0.20 < small < 0.50 < medium < 0.80 < large). For multiple-category variables, one-way ANOVAs were used, and categories with a different superscript letter show a significant difference between them in the psychological impact variable mean. In these cases, the effect size was assessed via η^2^ (interpretation: negligible < 0.01 < small < 0.06 < medium < 0.14 < large). ^1^Homoscedascity could not be assumed for these variables and thus the *t*-test results adjusted for non-homogeneous variances were used, and in the case of ANOVAS, *post hoc* Games-Howell tests were used.

### Contact With the COVID-19 Disease

Only 11 people in the sample had taken the COVID-19 test. Of them, eight received negative results and three positive results. Table 7 shows the frequency and percentage of participants who know someone with COVID-19 (31.5%), have had close contact in the previous 14 days with someone diagnosed with COVID-19 (9.5%), have had close contact with someone with COVID-19 symptoms or (suspected) infected material (24.2%), and have showed COVID-19 symptoms themselves (6.5%). Participants who have had close contact with someone diagnosed COVID-19 showed significantly higher psychological impact, stress, and anxiety that those who did not. Respondents who knew someone diagnosed with COVID-19 and those who have had close contact with someone with symptoms showed slightly higher stress and anxiety than those who did not. Participants with COVID-19 symptoms showed higher psychological distress in all the variables. All the effect sizes were at best small.

**TABLE 7:** Association between the degree of contact with the COVID-19 disease and the psychological impact of the COVID-19 pandemic as well as mental health status during the pandemic (*N* = 3055).

Variables	*N* (%)	Impact of event				Stress				Anxiety				Depression			
					
		*M* (*SD*)	*t*	*p*	g*	*M* (*SD*)	*t*	*p*	*g*	*M* (*SD*)	*t*	*p*	*g*	*M* (*SD*)	*t*	*p*	*g*
**Know someone with COVID-19**																	
No	2094 (68.5)	27.51 (19.13)	−1.85	0.07	0.07	10.73 (9.95)	−0.2.55	0.01	0.10	6 (8.01)	−2.15^1^	0.03	0.09	9.90 (10.04)	0.160	0.87	0.01
Yes	961 (31.5)	28.90 (19.37)				11.72 (10.20)				6.71 (8.57)				9.84 (9.86)			
**Close contact with someone diagnosed COVID-19**																	
No	2764 (90.5)	27.71 (19.09)	−2.03^1^	0.04	0.13	10.82 (9.93)	−3.45^1^	<0.01	0.23	6.06 (8.06)	−3.18^1^	<0.01	0.22	9.76 (9.90)	−1.91^1^	0.06	0.13
Yes	291 (9.5)	30.23 (20.27)				13.10 (10.78)				7.84 (9.23)				11.01 (10.68)			
**Close contact with someone with COVID-19 symptoms or (suspected) infected material**																	
No	2316 (75.8)	27.77 (19.34)	−0.90	0.37	0.04	10.71 (10.03)	−3.19	<0.01	0.21	6.05 (8.14)	−2.07	0.04	0.09	9.76 (10.03)	−1.22	0.22	0.05
Yes	739 (24.2)	28.50 (18.82)				12.07 (10)				6.77 (8.34)				10.27 (9.82)			
**Had COVID-19 symptoms**																	
No	2855 (93.5)	27.76 (19.21)	−2.10	0.04	0.15	10.86 (10.04)	−3.78	<0.001	0.28	6.03 (8.12)	−4.90	<0.001	0.36	9.73 (9.95)	−3.25	<0.01	0.24
Yes	200 (6.5)	30.71 (19.98)				13.63 (9.62)				8.96 (8.74)				12.09 (10.13)			

^1^Homoscedascity could not be assumed for these variables and thus the *t*-test results adjusted for non-homogeneous variances were used. * *g* = Hedges’ *g* effect size statistic. Interpretation: negligible < 0.20 < small < 0.50 < medium < 0.80 < large.

### Actual and Perceived Severity of the Situation

The average perceived severity of the COVID-19 outbreak in Spain was 8.49 (*SD* = 1.24; range 0–10). Perceived severity was significantly associated to psychological impact of the event (Spearman’s rho, ρ = 0.22; *p* < 0.001), stress (ρ = 0.13; *p* < 0.001), anxiety (ρ = 0.14; *p* < 0.001), and —very weakly— with depression (ρ = 0.07; *p* < 0.001). Concerning actual severity, by 17th March 2020, the day in which the data collection started (2 days after the state of alarm was implemented by Spanish Government) there were 11,273 cases and 497 deaths by COVID-19 in Spain. The last day of the data collection (24th March) there were 39,673 cases and 2,696 deaths. The actual severity of the situation showed a small though significant relation to perceived severity; the association between the number of infected people the day that each participant filled the questionnaires and his/her perceived severity was 0.10 (*p* < 0.001), and the association with the number of deaths was 0.09 (*p* < 0.001). The number of infected and death people by COVID-19 the day that the participant filled the questionnaire was unrelated to psychological impact, anxiety, or depression. There was only a significant and very weak association between number of infected people and stress (*r* = 0.04; *p* = 0.04).

### Information About the COVID-19 Pandemic

The most frequently used sources of information about the COVID-19 situation was TV (the main source for 40.7% of the participants) followed by social media (24.6%) and written press (20.1%). In general terms, participants whose main source of information was the radio showed lower distress than participants who preferably used TV, social media and written press. Respondents who indicated that they needed more information about the current situation (44.2%) showed poorer mental health. Participants who expressed being “somewhat satisfied” or “very satisfied” with the information received about the COVID-19 crisis showed lower distress than those who were somewhat or very dissatisfied. As for hours getting information about the COVID-19 situation in the last 24 h, 42.8% of the participants recognized having spent more than 2 h. Participants who spent three or more hours (21.8%) getting informed showed higher psychological impact, anxiety and depression than those who spent less time in this task (see Table 8). All effect sizes were at best small.

**TABLE 8:** Association between variables related to information about the COVID-19 pandemic and the psychological impact of the COVID-19 pandemic as well as mental health status during the pandemic (*N* = 3055).

Variables	*N* (%)	Impact of event				Stress				Anxiety				Depression			
					
		*M* (*SD*)	*t*/F*	*p*	*g*/η^2^*	*M* (*SD*)	*t*/F*	*p*	*g*/η^2^*	*M* (*SD*)	*t*/F*	*p*	*g*/η^2^*	*M* (*SD*)	*t*/F*	*p*	*g*/η^2^*
**Main source of information**																	
TV	1244 (40.7)	28.80 (19.55)^a^	3.08^1^	<0.01	0.01	10.87 (10.08)^a^	2.49^1^	0.02	0.01	6.27 (8.19)^a^	2.53^1^	0.01	0.01	9.91 (10.05)^ad^	4.81^1^	<0.001	0.01
Written press (digital/paper)	614 (20.1)	27.30 (19.68)^ab^				11.67 (10.67)^a^				6.34 (8.88)^a^				9.62 (10.03)^ad^			
Radio	98 (3.2)	22.70 (17.66)^b^				7.90 (7.41)^b^				3.41 (6.39)^b^				6.67 (7.48)^bc^			
Social media	751 (24.6)	29.08 (19.02)^a^				11.59 (9.78)^a^				6.67 (7.99)^a^				11.20 (10.37)^a^			
Messaging apps	165 (5.4)	24.84 (16.37)^ab^				9.96 (9.50)^ab^				6.12 (8.07)^ab^				8.35 (8.97)^cd^			
Family, friends or coworkers	116 (3.8)	25.98 (18.28)^ab^				10.29 (9.80)^ab^				4.78 (6.62)^ab^				8.62 (9.42)^abc^			
Official sources	30 (1)	23.00 (16.95)^ab^				11.13 (11.43)^ab^				6.60 (8.92)^ab^				8.20 (8.98)^abc^			
No specific source or other	37 (1.2)	25.00 (20.32)^ab^				10.38 (9.43)^ab^				5.95 (7.80)^ab^				7.03 (6.66)^cd^			
**Need for more information**																	
No	1706 (55.8)	26.34 (18.44)	−5.18^1^	<0.001	0.19	10.49 (9.67)	−3.36^1^	<0.01	0.12	5.47 (7.53)	−5.67^1^	<0.001	0.21	9.46 (9.65)	−2.60^1^	0.01	0.10
Yes	1349 (44.2)	29.99 (19.98)				11.73 (10.44)				7.18 (8.87)				10.41 (10.36)			
**Satisfaction with information**																	
Very dissatisfied	355 (11.6)	31.82 (20.94)^a^	16.59^1^	<0.001	0.02	12.74 (11.07)^a^	19.10^1^	<0.001	0.02	7.95 (9.69)^a^	16.25^1^	<0.001	0.02	11.33 (11.12)^a^	10.74^1^	<0.001	0.01
Somewhat dissatisfied	884 (28.9)	30.14 (19.64)^a^				12.52 (10.30)^a^				6.98 (8.38)^a^				10.77 (10.20)^a^			
Somewhat satisfied	1358 (44.5)	26.81 (18.71)^b^				10.35 (9.69)^b^				5.90 (8.04)^b^				9.55 (9.81)^b^			
Very satisfied	458 (15)	24.11 (17.39)^c^				8.913 (9.03)^c^				4.41 (6.44)^c^				8.02 (8.70)^c^			
**Hours getting information (last 24 h)**																	
Less than 1h	583 (19.1)	25.54 (18.56)^a^	11.80^1^	<0.001	0.02	9.97 (9.60)^a^	6.23^1^	<0.001	0.01	5.46 (7.94)^a^	7.72^1^	<0.001	0.01	9.17 (9.41)	2.83^1^	0.02	0
1–2 h	1163 (38.1)	26.62 (18.51)^a^				10.66 (9.72)^ac^				5.73 (7.71)^a^				9.67 (9.79)			
2–3 h	643 (21)	28.26 (18.61)^ab^				11.06 (9.69)^abc^				6.36 (8.26)^ab^				9.80 (9.80)			
3–5 h	385 (12.6)	31.52 (19.87)^bc^				12.64 (10.88)^bd^				7.29 (8.71)^b^				10.86 (10.75)			
Over 5h	281 (9.2)	32.86 (22.20)^c^				12.58 (11.35)^cd^				8.09 (9.34)^b^				11.08 (11.02)			

* Differences in mean level between categories of dichotomous variables were assessed via *t*-test and Hedges’ *g* effect size statistic was obtained (interpretation: negligible < 0.20 < small < 0.50 < medium < 0.80 < large). For multiple-category variables, one-way ANOVAs were used, and categories with a different superscript letter show a significant difference between them in the psychological impact variable mean. In these cases, the effect size was assessed via η^2^ (interpretation: negligible < 0.01 < small < 0.06 < medium < 0.14 < large). ^1^Homoscedascity could not be assumed for these variables and thus the *t*-test results adjusted for non-homogeneous variances were used, and in the case of ANOVAS, *post hoc* Games-Howell tests were used.

### Health Status

The mean for perceived health status (0–10) was 7.77 (*SD* = 1.51). There were no significant differences in perceived health level when the different age groups were compared (*p* = 0.053). Higher perceived level of health was associated to lower psychological impact (Spearman’s rho, ρ = −0.19, *p* < 0.001), stress (ρ = −0.25, *p* < 0.001), anxiety (ρ = −0.27, *p* < 0.001), and depression (ρ = −0.27, *p* < 0.001). Participants who considered themselves part of the high-risk population in case of being infected with COVID-19 (19.1%) reported significantly higher levels of psychological impact, anxiety, stress and depression symptoms than those who thought that were not in the high-risk population group. Those who reported having suffered any of the symptoms of COVID-19 (33.3%), sore throat (25.1%), headache (43.5%), muscle or joint pain (18.7%), cough (28.30%), fatigue (15.2%), and shortness of breath (9.3%) scored higher in all the variables. The differences were especially significant for shortness of breath. A total of 171 participants (5.6%) had called the COVID-19 hotline and showed significantly higher levels of psychological distress (*p* < 0.05 in all the variables) (see Supplementary Table 1). All effect sizes were small except for fatigue, with a moderate effect size on stress and anxiety, and shortness of breath, with a moderate effect on psychological impact, stress, and depression and a large effect on anxiety.

### Leisure Activities During the Home Confinement

Most frequent leisure activities during confinement were talking to someone via telephone, instant messaging or videocalls (96.8%), browsing or sharing social network contents (85.2%), watching films or shows (85%), watching TV (79.1%), reading (52.4%), and practicing sports or physical exercise (48.7%). The total number of leisure activities in which participants had engaged during the previous 24 h was computed, and its correlation with psychological distress was calculated. Correlations were negative and significant for stress (*r* = −0.11; *p* < 0.001), anxiety (*r* = −0.11; *p* < 0.001), depression (*r* = −0.14; *p* < 0.001), and —although weaker— for psychological impact of the event (*r* = −0.05; *p* < 0.001). We compared the levels of distress of those who had engaged on each leisure activity and those who had not. Practicing physical activity and/or watching films or shows were associated to lower stress, anxiety and depression scores. Reading and making handicrafts or art activities were related to lower scores in all the variables (see Supplementary Table 2). All effect sizes were small at best.

## Discussion

The COVID-19 pandemic is a global health threat. As of 9th April, Spain is the second country in confirmed cases of infected people (146,690) and the third in number of deaths (14,555; European Centre for Disease Prevention and Control, 2020). As COVID-19 is transmitted from one person to another, several Governments, including the Spanish, have implemented extreme restriction measures to people’s movements in order to ameliorate the virus spread. The uncertainty of how this new illness will develop together with the unusual situation of being confined at home is most likely leading Spaniards to suffer negative psychological consequences (Brooks et al., 2020; Wang et al., 2020). Despite the urgent need claimed by several authors to systematically examine the psychological health of the population being most affected by the COVID-19 pandemic (Brooks et al., 2020; de Carvalho et al., 2020; Duan and Zhu, 2020; Zandifar and Badrfam, 2020), scientific data on this matter is so far scarce. To fill this gap in the literature, this study focused on the psychological impact that the first stages of COVID-19 crisis had on Spanish psychological health. Specifically, we collected data on the psychological impact of the COVID-19 crisis on adult Spaniards’ mental health, including psychological impact in terms of symptomatic responses (avoidance, intrusion, and hyperarousal), as well as stress, anxiety, and depression symptoms.

Our results showed that most participants had experienced significant life changes due to this health crisis. These include changes in the financial and/or work situation, a severe restriction in movements and cancelation of important activities. All these took place in a very short period of time and, consequently, our findings show that Spaniards perceived the current situation to be quite severe. Regarding the effects of the health crisis on the Spanish population, 63% the participants reported minimal to mild acute stress symptoms during the initial stage of the pandemic outbreak, a number that paints a more favorable picture than data from China, where about 45% fell into that category (Wang et al., 2020). Nevertheless, over a third of Spaniards showed symptoms of moderate or severe psychological impact, a number below China’s 54% (Wang et al., 2020), though still worrying. Concerning stress and depression, Spaniards showed moderate to severe levels to a higher degree (22 and 30%, respectively) than Chinese participants (8 and 17%). Lastly, regarding anxiety, Spanish (24%) showed similar levels to those of the Chinese population (29%). In the current study, participants’ perceived health level was negatively associated with psychological impact, stress, anxiety, and depression symptoms. This means that perceptions of the severity of the situation were more strongly associated with subsequent negative psychological impact than objective aspects of the experience. In fact, those who had experienced symptoms that could be related to COVID-19, such as cough or shortness of breath, showed poorer psychological health, although they did now know whether they were infected. This relation was stronger for those who considered to be part of the high-risk population. Interestingly, perceived health level was not related to age, which suggests a stronger need to pay special attention to those who perceived themselves as vulnerable, despite their actual risk. These results suggest that, in line with previous studies (Brooks et al., 2020; Liu et al., 2020), the uncertainty of the health situation as well as its development and consequences can lead to suffering from stress, anxiety, and depression even when showing just mild (maybe) related COVID-19 symptoms, such as cough. Taken together, these results highlight the great negative psychological impact that the COVID-19 pandemic is having on the population in the early stages of the outbreak, although it must be kept in mind that most effect sizes were small. These numbers could also signal toward the future development of negative psychological outcomes that are common in the aftermath of crises and disasters, such as posttraumatic stress disorder, generalized anxiety or major depression disorders, and substance abuse (Boscarino, 2015; Mazumder, 2015).

In accordance with other studies carried out in China about the COVID-19 pandemic (Liu et al., 2020; Sun et al., 2020; Wang et al., 2020), women and young adults were the ones that suffered the greater psychological impact, though again we must remind the reader about the small effect sizes in most cases. This result should not come as a surprise if we consider the ways that gender roles differentially affect women and men (Wenham et al., 2020). For instance, many of the industries most affected by the COVID-19 health crisis employ mostly women, who consequently are at higher risk of job and income loss (Ramos, 2020). Moreover, women are usually the informal caregivers within families, so the necessary restrictive measures, such as schools and childcare facilities closures, increase their burden at home (Mantovani et al., 2020; Ramos, 2020). This can substantially reduce women’s ability to perform their work duties, whether they are working from home or on site (Gausman and Langer, 2020). This leads women to experience more difficulties to keep their job, limiting their work opportunities and financial status (Wenham et al., 2020). Women also constitute the majority of health-care workforce, therefore being more likely to be infected by the coronavirus (Wenham et al., 2020) and to put their families at risk. It should also be noted that higher rates of domestic violence against women are usually registered during times of crisis and quarantines (Gausman and Langer, 2020; Ramos, 2020), which constitutes another source of distress. Our results can contribute to the understanding of gendered impacts of disease outbreaks identified not only for the current COVID-19 but for past outbreaks such as Ebola or the Zika virus (Wenham et al., 2020). This is fundamental to comprehend the primary and secondary effects of health emergencies as well as to design interventions that fit the patients’ needs.

As for age, some literature in the field of disaster indicates that the elderly are particularly vulnerable to the negative psychological sequelae of critical situations, such as PTSD (Jia et al., 2010). However, in line with our results, most of the studies have found that age constitutes a protective effect that in our case had a medium effect size. Older disaster victims usually show lower stress, anxiety, and depression symptoms than younger participants, and this trend may be explained by their greater life experience, previous disaster exposure or by having to face fewer life responsibilities (see Ngo, 2001 for a review). Future studies should explore the psychological impact of the COVID-19 pandemic in a larger sample of elderly population, and whether younger and older participants recover differently from the psychological sequelae of the COVID-19 crisis.

Being married or cohabiting with partner was a protective factor against psychological suffering with a medium effect size, as has usually been found in the literature (Frech and Williams, 2007; Kalmijn, 2017), while being in a relationship but not cohabiting was an important risk factor, also in line with research reporting on the positive effects of cohabiting (e.g., Kalmijn, 2017). For people in relationships but not cohabiting, the home confinement situation may resemble that of a long-distance relationship, which studies have linked to increased individual and relationship stress (Du Bois et al., 2016) and to possible disruption of psychobiological linkage between partners (Diamond, 2019). Since technology-mediated communications have proved beneficial in separated couples (Tong and Walther, 2011; Carter and Renshaw, 2016), these should be an obvious recommendation to alleviate the impact of the health crisis. Interestingly, having children appeared to be a protective factor against psychological suffering, although one of a small effect size. One could have expected that being confined at home with children leads to higher levels of anxiety and stress, especially to those who have to work from home while taking care of their children. Our data showed otherwise, in line with results from studies showing that parenthood increases subjective well-being (Nomaguchi, 2012; Radó, 2019), which appears to be the case even in the extreme circumstances of the COVID-19 health crisis. Also related to people cohabiting, we found that the lower the house population density, the better the mental health, with a small effect size. This is in line with previous studies showing the negative impact of crowding on mental health and psychological functioning (Evans and Wener, 2007; Thornock et al., 2020). It remains to be explored how long-term confinement at home impacts in the relationship with cohabitants, given that conflicts may be enhanced by this unusual and potentially stressful situation (Mesa Viera et al., 2020). Our results show that the negative psychological impact of the lockdown increase as the days pass by. Thus, in accordance Brooks et al. (2020), we recommend that quarantine should last no longer that necessary and information about the rationale of this very restrictive measure as well as of the positive effects that it has in this health crisis should be regularly provided.

Similar to Wang et al.’s (2020) results, lower educational level and family income were associated with stronger negative psychological effects. Being employed was linked to better mental health. More than 12% of employed participants had been forced to stop working altogether or had lost their job during the first days of national lockdown, a number that can only be expected to increase as the crisis develops. People who had lost their job or had stopped working during the health crisis and those who were working on site reported the highest levels of psychological impact, stress, anxiety, and depression. This result points to the significant challenge created by this crisis on an organizational level, where the most favorable outcome for people would be to keep their job and work remotely from home. Again, the small sizes of the effects must be considered. In line with the importance of work-related variables and economic stability for keeping mental health in times of crisis, it is worth noticing that what worried Spaniards the most was not health-related, but had to do with the economic recession that most likely will follow the current health crisis. This is only normal considering that the mental health problems related to the 2008 financial and banking crisis, which was especially hard in Spain, are still present in the Spanish population (Iglesias-García et al., 2017; Rivera et al., 2017). In fact, according to our findings, many people are in fear of losing their job and/or suffering a decrease in their family income. Thus, if we want to prevent a great deal of long-lasting psychological suffering for Spanish and people in other countries experiencing a similar situation, the urgent call made by some European governments to look for a united approach to deal with the upcoming economic recession should be seriously considered.

Our data correspond to the first week of home confinement in Spain, and so the results only provide information about the population’s mental health at the beginning of the health crisis, which may explain the generally small effect sizes found. The COVID-19 pandemic is still ongoing and the psychological consequences derived from this health emergency (Liu et al., 2020; Sun et al., 2020; Wang et al., 2020) are likely to have a lasting effect long after the pandemic is under control, which should be explored in future longitudinal studies (Brooks et al., 2020). Such studies might find larger effect sizes. Hence, there is an urgent need of psychological interventions aimed at ameliorating the negative psychological impact of the COVID-19 (Duan and Zhu, 2020; Xiang et al., 2020). Our findings have implications for the design of such psychological interventions. We believe interventions should be provided in two different moments (Zhang et al., 2020). First, during the outbreak, so that the psychological negative effect of the health crisis can be ameliorated and the expected increase in these symptoms as the lockdown continues can be diminished. This will most likely help people to cope with and adapt to the current situation, lowering the risk of suffering future psychopathologies (de Carvalho et al., 2020).

A first step toward psychological interventions during the outbreak is through mass media. Our findings —although the small effect sizes must again be considered— suggest that people who are more satisfied with the information received about COVID-19 show the lower psychological distress, as well as those who spent no more than 3 h per day getting informed. Thus, it is necessary to help people look for information only in official sources by, for instance, clearly indicating them on TV, radio, and newspapers. Another recommendation would be to not rely on social networks and the TV as the main source of information, in favor of the radio. It is also necessary to give the general public some specific guidelines to follow during the lockdown so that they can take care of their mental health. This include investing their time in leisure activities (Brooks et al., 2020) that will most likely keep their mind busy and, thus, minimize rumination (Hilt and Pollak, 2012). Moreover, physical activity has been seen to improve people’s mood (Penedo and Dahn, 2005) and is a good strategy to cope with the downsides of confinement (Brooks et al., 2020). Finally, when the person feels that they cannot cope with the negative psychological symptoms derived from the current health crisis, online-based therapy can be a good option (Abbott et al., 2008).

Second, interventions should also be provided once the situation progressively goes back to normal. Considering that PTSS can remain a long time after the event took place (Neria et al., 2011) or even occur with delayed onset (Smid et al., 2009; Utzon-Frank et al., 2014), and that the same applies to depression symptoms (Bonde et al., 2016), mental health experts should be prepared to deliver therapeutic interventions with those who will psychologically suffer from the current health crisis in the upcoming years. Additionally, in the case that new secondary outbreaks of COVID-19 occur in the future, it seems crucial to explore their psychological impact.

This study is not without limitations. First, we followed a snowball sampling technique. This was quite successful, leading to a sample of more than 3,000 participants, but it has some downsides. There was an oversampling of people living in Madrid. The questionnaire was launched national-wide but, at the time of the data collection, the COVID-19 outbreak was more severe in Madrid. This might have motivated people living in that province, as compared to residents from other regions, to fill the questionnaire. We also count with a large sample of young participants, while only 2% of the participants were 65 years old or over. This may probably be explained by the way the questionnaire was disseminated. Due to the state of alarm, dissemination was done through social media technologies (i.e., WhatsApp, Twitter, and Instagram). This required the use of information and communication technologies, which is less common for older people. In addition, more women than men participated in the study, coherently with previous research acknowledging that it is more difficult to recruit male than female participants (Korkeila et al., 2001; Dunn et al., 2004), and variable distribution shape might differ between this sample and the population, which is why the findings of this study should only be generalized with caution. Second, the present study reports on data on the early stages of the COVID-19 pandemic in Spain and most of the effect sizes were small. Consequently, results should be taken with caution and future studies should further explore the relative contribution of these variables at later stages of the health crisis, when its effects might be more prominent. Third, as already noted our aim was to provide a clear picture of the psychological impact of the pandemic in the Spanish population on its early stages. Considering the lack of tests to check whether a person was infected with COVID-19 during these first weeks of the outbreak, it is normal that only eleven of the 3,055 participants were tested for COVID-19 and the result was negative for most of them. These people showed much lower negative psychological consequences of the pandemic than the rest, but results need be taken with caution as this small subsample cannot be seen as representative. A second data collection conducted a few weeks after the state of alarm declaration may reveal whether these results can be generalized. As for the variables included in this research, more recently discovered COVID-19 markers such as loss of smell and taste (Menni et al., 2020) should be added in future studies exploring the associations between COVID-19 symptomatology and psychological impact.

## Conclusion

The COVID-19 pandemic has negative psychological effects on Spanish people. Those who suffer the most are women, young people, and those who consider themselves to be in the risk-population group. Our findings can help design such interventions so that people who have seen their psychological health diminished during the pandemic can better cope with this difficult situation, both in Spain and other parts of the world. Considering this current health crisis will most likely have long lasting effects (Liu et al., 2020; Sun et al., 2020), follow-up studies are needed to obtain a clear picture of the magnitude of the psychological impact of COVID-19 pandemic.

## Data Availability Statement

The raw data supporting the conclusions of this article will be made available by the authors, without undue reservation.

## Ethics Statement

The studies involving human participants were reviewed and approved by Comité de Ética de la Universidad Pontificia Comillas y Comité de Ética del Centro Universitario Cardenal Cisneros. The patients/participants provided their written informed consent to participate in this study.

## Author Contributions

RR-R, HG-H, and SC conceptualized and designed the study, and were involved in data collection. RR-R and HG-H performed the data analyses. RR-R prepared the first draft of the manuscript. HG-H and SC revised and improved the quality of the analyses performed, critically revised the draft, and made important contributions. All the authors read and approved the final version of the manuscript.

## Conflict of Interest

The authors declare that the research was conducted in the absence of any commercial or financial relationships that could be construed as a potential conflict of interest.
