# Supplementary material for: Psychological Impact and Associated Factors During the Initial Stage of the Coronavirus (COVID-19) Pandemic Among the General Population in Spain
Source: Front Psychol. 2020 Jun 23;11:1540. doi: 10.3389/fpsyg.2020.01540 (PMC7325630; doi:10.3389/fpsyg.2020.01540)
Supplement: Supplementary file 1 [file Table_1.docx]

Supplementary Table S1. Association between health status and the psychological impact of the COVID-19 pandemic as well as mental health status during the pandemic.

| **Variables** |  | **Impact of event** | | | | **Stress** | | | | **Anxiety** | | | | **Depression** | | | |
| --- | --- | --- | --- | --- | --- | --- | --- | --- | --- | --- | --- | --- | --- | --- | --- | --- | --- |
|  | ***N* (%)** | *M* (*SD*) | *t* | *p* | *g* * | *M* (*SD*) | *t* | *p* | *g* | *M* (*SD*) | *t* | *p* | *g* | *M* (*SD*) | *t* | *p* | *g* |
| **High risk population** | | | | | | | | | | | | | | | | | |
| No | 2470 (80.9) | 27.52 (18.81) | -2.37 ^1^ | .02 | .12 | 10.85 (9.84) | -2.20 ^1^ | .03 | .11 | 5.87 (7.81) | -4.32 ^1^ | < .001 | .23 | 9.71 (9.71) | -1.72 ^1^ | .09 | .09 |
| Yes | 585 (19.1) | 29.75 (20.78) |  |  |  | 11.91 (10.77) |  |  |  | 7.71 (9.53) |  |  |  | 10.57 (11.01) |  |  |  |
| **Fever** | | | | | | | | | | | | | | | | | |
| No | 2948 (96.5) | 27.96 (19.19) | .12 | .90 | .01 | 11.01 (10.04) | -.85 | .40 | .08 | 6.17 (8.16) | -1.87 | .06 | .18 | 9.85 (9.97) | -.76 | .45 | .08 |
| Yes | 107 (3.5) | 27.73 (20.09) |  |  |  | 11.85 (10.05) |  |  |  | 7.68 (9.12) |  |  |  | 10.60 (10.21) |  |  |  |
| **Sore throat** | | | | | | | | | | | | | | | | | |
| No | 2289 (74.9) | 26.96 (19.06) | -4.92 | < .001 | .21 | 10.54 (10.03) | -4.72 | < .001 | .20 | 5.71 (7.90) | -5.77 ^1^ | < .001 | .25 | 9.49 (9.77) | -3.63 ^1^ | < .001 | .16 |
| Yes | 766 (25.1) | 30.89 (19.38) |  |  |  | 12.52 (9.91) |  |  |  | 7.78 (8.83) |  |  |  | 11.05 (10.49) |  |  |  |
| **Headache** | | | | | | | | | | | | | | | | | |
| No | 1727 (56.5) | 25.43 (18.46) | -8.31 ^1^ | < .001 | .31 | 9.41 (9.55) | -10.28 ^1^ | < .001 | .38 | 4.88 (7.29) | -10.26 ^1^ | < .001 | .38 | 8.51 (9.27) | -8.58 ^1^ | < .001 | .32 |
| Yes | 1328 (43.5) | 31.24 (19.68) |  |  |  | 13.15 (10.26) |  |  |  | 7.97 (8.94) |  |  |  | 11.66 (10.57) |  |  |  |
| **Muscle or joint pain** | | | | | | | | | | | | | | | | | |
| No | 2484 (81.3) | 27.10 (18.82) | -4.85 ^1^ | < .001 | .24 | 10.31 (9.72) | -7.95 ^1^ | < .001 | .39 | 5.63 (7.80) | -7.58 ^1^ | < .001 | .39 | 9.40 (9.68) | -5.15 ^1^ | < .001 | .26 |
| Yes | 571 (18.7) | 31.64 (20.46) |  |  |  | 14.21 (10.75) |  |  |  | 8.81 (9.31) |  |  |  | 11.96 (10.94) |  |  |  |
| **Cough** | | | | | | | | | | | | | | | | | |
| No | 2188 (71.62) | 27.51 (19.62) | -2.07 ^1^ | .04 | .08 | 10.69 (10.18) | -3.16 ^1^ | < .01 | .12 | 5.86 (8.20) | -3.97 | < .001 | .16 | 9.47 (9.94) | -3.60 | < .001 | .14 |
| Yes | 867 (28.38) | 29.05 (18.12) |  |  |  | 11.93 (9.60) |  |  |  | 7.16 (8.12) |  |  |  | 10.91 (10.00) |  |  |  |
| **Shortness of breath** | | | | | | | | | | | | | | | | | |
| No | 2772 (90.7) | 26.98 (18.90) | -8.85 | < .001 | .55 | 10.43 (9.83) | -10.75 | < .001 | .67 | 5.48 (7.59) | -13.00 ^1^ | < .001 | 1.02 | 9.35 (9.70) | -8.28 ^1^ | < .001 | .58 |
| Yes | 283 (9.3) | 37.46 (19.71) |  |  |  | 17.04 (10.09) |  |  |  | 13.53 (10.12) |  |  |  | 15.03 (11.15) |  |  |  |
| **Fatigue** | | | | | | | | | | | | | | | | | |
| No | 2591 (84.8) | 26.88 (18.71) | -6.78 ^1^ | < .001 | .37 | 10.26 (9.70) | -9.58 ^1^ | < .001 | .52 | 5.52 (7.67) | -9.77 ^1^ | < .001 | .58 | 9.16 (9.55) | -8.56 ^1^ | < .001 | .48 |
| Yes | 464 (15.2) | 33.91 (20.87) |  |  |  | 15.38 (10.74) |  |  |  | 10.19 (9.77) |  |  |  | 13.91 (11.27) |  |  |  |
| **Lack of symptoms** | | | | | | | | | | | | | | | | | |
| No | 2039 (66.7) | 30.24 (19.35) | 9.66 ^1^ | <.001 | .36 | 12.50 (10.11) | 12.01 ^1^ | < .001 | .45 | 7.46 (8.66) | 13.31 ^1^ | < .001 | .46 | 11.10 (10.35) | 10.27 ^1^ | < .001 | .37 |
| Yes | 1016 (33.3) | 23.36 (18.10) |  |  |  | 8.11 (9.22) |  |  |  | 3.74 (6.51) |  |  |  | 7.44 (8.70) |  |  |  |
| **Phone call to COVID-19 hotline** | | | | | | | | | | | | | | | | | |
| No | 2884 (94.4) | 27.70 (19.11) | -2.97 | < .01 | .23 | 10.87 (10.00) | -3.77 | < .001 | .30 | 6.04 (8.05) | -4.37 ^1^ | < .001 | .41 | 9.74 (9.93) | -3.28 | < .01 | .26 |
| Yes | 171 (5.6) | 32.19 (20.52) |  |  |  | 13.85 (10.27) |  |  |  | 9.38 (9.77) |  |  |  | 12.30 (10.48) |  |  |  |

^1^ Homoscedascity could not be assumed for these variables and thus the *t*-test results adjusted for non-homogeneous variances were used.

* *g* = Hedges’ *g* effect size statistic. Interpretation: negligible < .20 < small < .50 < medium < .80 < large.
