# Supplementary material for: Psychological Impact and Associated Factors During the Initial Stage of the Coronavirus (COVID-19) Pandemic Among the General Population in Spain
Source: Front Psychol. 2020 Jun 23;11:1540. doi: 10.3389/fpsyg.2020.01540 (PMC7325630; doi:10.3389/fpsyg.2020.01540)
Supplement: Supplementary file 2 [file Table_2.docx]

Supplementary Table S2. Association between leisure activities in the last 24 hours and the psychological impact of the COVID-19 pandemic as well as mental health status during the pandemic (*N* = 3055)

|  | **N (%)** | **Impact of event** | | | | **Stress** | | | | **Anxiety** | | | | **Depression** | | | |
| --- | --- | --- | --- | --- | --- | --- | --- | --- | --- | --- | --- | --- | --- | --- | --- | --- | --- |
|  |  | *M (SD)* | *t* | *p* | *g* * | *M (SD)* | *t* | *p* | *g* | *M (SD)* | *t* | *p* | *g* | *M (SD)* | *t* | *p* | *g* |
| **Sport or physical exercise** | | | | | | | | | | | | | | | | |  |
| No | 1568 (51.3) | 28.23 (19.18) | .81 | .42 | .03 | 11.75 (10.50) | 4.04 ^1^ | < .001 | .15 | 6.80 (8.38) | 4.00 ^1^ | < .001 | .15 | 10.98 (10.60) | 6.32 ^1^ | < .001 | .23 |
| Yes | 1487 (48.7) | 27.66 (19.25) |  |  |  | 10.29 (9.46) |  |  |  | 5.61 (7.95) |  |  |  | 8.72 (9.13) |  |  |  |
| **Watching films or shows** | | | | | | | | | | | | | | | | |  |
| No | 458 (15) | 28.69 (18.89) | .90 | .37 | .05 | 12.31 (10.63) | 2.79 ^1^ | < .01 | .15 | 7.09 (8.83) | 2.29 ^1^ | .02 | .12 | 11.00 (10.67) | 2.45 ^1^ | < .01 | .13 |
| Yes | 2597 (85) | 27.82 (19.27) |  |  |  | 10.82 (9.91) |  |  |  | 6.07 (8.07) |  |  |  | 9.68 (9.84) |  |  |  |
| **Reading** | | | | | | | | | | | | | | | | |  |
| No | 1455 (47.6) | 29.15 (19.12) | 3.31 | < .01 | .12 | 11.91 (10.31) | 4.55 ^1^ | < .001 | .17 | 6.89 (8.50) | 4.27 ^1^ | < .001 | .16 | 11.16 (10.42) | 6.76 ^1^ | < .001 | .25 |
| Yes | 1600 (52.4) | 26.86 (19.24) |  |  |  | 10.25 (9.72) |  |  |  | 5.62 (7.86) |  |  |  | 8.72 (9.41) |  |  |  |
| **Watching TV** | | | | | | | | | | | | | | | | |  |
| No | 639 (20.9) | 28.15 (19.49) | .29 | .77 | .01 | 12.07 (10.77) | 2.77 ^1^ | < .01 | .13 | 6.93 (8.71) | 2.34 ^1^ | .02 | .11 | 10.76 (10.84) | 2.35 ^1^ | .02 | .11 |
| Yes | 2416 (79.1) | 27.90 (19.15) |  |  |  | 10.77 (9.82) |  |  |  | 6.04 (8.05) |  |  |  | 9.65 (9.73) |  |  |  |
| **Handicrafts or art activities** | | | | | | | | | | | | | | | | |  |
| No | 2212 (72.4) | 28.46 (19.35) | 2.38 | .02 | .10 | 11.36 (10.24) | 2.99 ^1^ | < .01 | .12 | 6.45 (8.35) | 2.97 ^1^ | < .01 | .11 | 10.37 (10.18) | 4.61 ^1^ | < .001 | .18 |
| Yes | 843 (27.6) | 26.61 (18.81) |  |  |  | 10.19 (9.44) |  |  |  | 5.54 (7.74) |  |  |  | 8.59 (9.31) |  |  |  |
| **Playing games** | | | | | | | | | | | | | | | | |  |
| No | 1553 (50.8) | 28.25 (19.24) | .89 | .37 | .03 | 11.36 (10.13) | 1.81 | .07 | .06 | 6.37 (8.34) | 1.03 | .31 | .04 | 10.00 (9.94) | .68 | .50 | .03 |
| Yes | 1502 (49.2) | 27.64 (19.19) |  |  |  | 10.71 (9.93) |  |  |  | 6.07 (8.04) |  |  |  | 9.75 (10.02) |  |  |  |
| **Browsing or sharing social network contents** | | | | | | | | | | | | | | | | |  |
| No | 451 (14.8) | 26.37 (20.31) | -1.90 | .06 | .10 | 10.44 (10.85) | -1.28 ^1^ | .20 | .07 | 6.39 (8.95) | .42 ^1^ | .68 | .02 | 9.95 (10.87) | .14 ^1^ | .89 | .01 |
| Yes | 2604 (85.2) | 28.22 (19.01) |  |  |  | 11.14 (9.89) |  |  |  | 6.20 (8.06) |  |  |  | 9.87 (9.82) |  |  |  |
| **Talking (phone, videocalls…)** | | | | | | | | | | | | | | | | |  |
| No | 98 (3.2) | 25.96 (21.98) | -1.04 | .30 | .11 | 10.06 (11.54) | -0.86 ^1^ | .39 | .10 | 7.45 (10.52) | 1.18 ^1^ | .24 | .15 | 11.76 (12.58) | 1.51 ^1^ | .14 | .19 |
| Yes | 2957 (96.8) | 28.01 (19.12) |  |  |  | 11.07 (9.98) |  |  |  | 6.18 (8.11) |  |  |  | 9.82 (9.88) |  |  |  |
| **Other leisure activities** | | | | | | | | | | | | | | | | |  |
| No | 2550 (83.5) | 28.31 (19.23) | 2.36 | .02 | .11 | 11.28 (10.06) | 2.91 | < .01 | .14 | 6.35 (8.28) | 2.04 ^1^ | .04 | .09 | 10.07 (9.97) | 2.35 | .02 | .11 |
| Yes | 505 (16.5) | 26.11 (19.05) |  |  |  | 9.85 (9.81) |  |  |  | 5.58 (7.71) |  |  |  | 8.93 (10.00) |  |  |  |

^1^ Homoscedascity could not be assumed for these variables and thus the *t*-test results adjusted for non-homogeneous variances were used.

* *g* = Hedges’ *g* effect size statistic. Interpretation: negligible < .20 < small < .50 < medium < .80 < large.
